# Effect of Cornstalk Biochar Immobilized Bacteria on Ammonia Reduction in Laying Hen Manure Composting

**DOI:** 10.3390/molecules25071560

**Published:** 2020-03-28

**Authors:** Huaidan Zhang, Jeremy N. Marchant-Forde, Xinyi Zhang, Yan Wang

**Affiliations:** 1 College of Animal Science, South China Agricultural University, Guangzhou 510642, China; zhd520888@163.com (H.Z.); zsz@stu.scau.edu.cn (X.Z.); 2 Livestock Behavior Research Unit, USDA-ARS, West Lafayette, IN 47907, USA; jeremy.marchant-forde@usda.gov; 3 Guangdong Provincial Key Lab of Agro-Animal Genomics and Molecular Breeding and Key lab of Chicken Genetics, Breeding and reproduction, Ministry of Agriculture, Guangzhou 510642, China

**Keywords:** cornstalk biochar immobilized mixed bacteria, ammonia, composting, laying hen manure, cornstalk biochar

## Abstract

NH_3_ emission has become one of the key factors for aerobic composting of animal manure. It has been reported that adding microbial agents during aerobic composting can reduce NH_3_ emissions. However, environmental factors have a considerable influence on the activity and stability of the microbial agent. Therefore, this study used cornstalk biochar as carriers to find out the better biological immobilization method to examine the mitigation ability and mechanism of NH_3_ production from laying hen manure composting. The results from different immobilized methods showed that NH_3_ was reduced by 12.43%, 5.53%, 14.57%, and 22.61% in the cornstalk biochar group, free load bacteria group, mixed load bacteria group, and separate load bacteria group, respectively. Under the simulated composting condition, NH_3_ production was 46.52, 38.14, 39.08, and 30.81 g in the treatment of the control, mixed bacteria, cornstalk biochar, and cornstalk biochar separate load immobilized mixed bacteria, respectively. The cornstalk biochar separate load immobilized mixed bacteria treatment significantly reduced NH_3_ emission compared with the other treatments (*p <* 0.05). Compared with the control, adding cornstalk biochar immobilized mixed bacteria significantly decreased the electrical conductivity, water-soluble carbon, total nitrogen loss, and concentration of ammonium nitrogen (*p <* 0.05), and significantly increased the seed germination rate, total number of microorganisms, and relative abundance of lactic acid bacteria throughout the composting process (*p <* 0.05). Therefore, the reason for the low NH_3_ emission might be due not only to the adsorption of the cornstalk biochar but also because of the role of complex bacteria, which increases the relative abundance of lactic acid bacteria and promotes the acid production of lactic acid bacteria to reduce NH_3_ emissions. This result revealed the potential of using biological immobilization technology to reduce NH_3_ emissions during laying hen manure composting.

## 1. Introduction

Egg production has been increasing worldwide [1,2,3], and to meet the needs of consumers, a large number of laying hens are farmed. This has produced a huge quantity of feces that require treatment [4,5]. In order to treat manure, current laying hen farms have mainly used high temperature aerobic composting, anaerobic fermentation, feed treatment, and direct return to the field [6]. Anaerobic fermentation can realize the harmless treatment of feces and reuse resources [7]. However, it cannot be widely used due to the high cost and geographical limitations. Although it is simple to return directly to the field, it is easy to cause pollution problems [8]. Feed treatment needs further research due to its safety [9]. High temperature aerobic composting has been widely used as an efficient, economical, and hygienic approach to fecal treatment for intensive laying hen farms [10,11].

During the composting process, organic matter in laying hen manure is continuously catabolized by microorganisms to produce metabolites, and some intermediate products and final products are volatile odor substances [12]. Odor in compost can be divided into four categories: Volatile nitrogen compounds, volatile sulfur compounds, volatile fatty acids, and aromatic compounds [13]. The emission of many types of malodorous gas in the composting process has become an obstacle to its development [14], and the high concentration of ammonia (NH_3_) needs to be addressed. According to the animal, especially poultry, health guidelines, ammonia should be kept below 25 ppm. Therefore, the high concentration of ammonia means severe environmental pollution and the loss of a large amount of nitrogen sources [15], normally around or over 25 ppm. Additionally, ammonia odors are the main reason for complaints about farms, and ammonia volatilization will lead to nitrogen loss and affect the nutrient value of the compost [16,17]. Research showed that ammonia volatilization was the primary cause of nitrogen loss in the compost, accounting for more than 90% of the total loss [18]. In addition, the reaction of ammonia with nitrogen oxides and sulfur dioxide in the air results in the deposition of nitrates and phosphates, which acidifies the ecosystem and catalyzes the formation of acid rain, which adversely affects the longevity of the composting equipment [19,20].

Adding microbial additives during the composting process is a significant measure to improve the quality of compost and reduce the release of NH_3_ [21]. Gutarowska et al. (2014) found that *Bacillus* subtilis subsp. *spizizenii, Leuconostoc mesenteroides, Candida inconspicua*, and *Psychrobacter faecalis*, had better deodorizing effects in laying hen manure, and the mixture’s removal rate of NH_3_ reached 20.8% [22]. Wang et al. (2019) added 8g/kg of *Bacillus stearothermophilus* to manure composting and found that the NH_3_ emission was reduced by 11.21% [23]. Our previous study found that adding a small amount (5%) of complex bacteria (*Bacillus stearothermophilus*, *Candida utilis*, and *Bacillus subtilis*) to a small simulated experimental study of laying hen manure compost could have a certain inhibitory effect on NH_3_, and the highest removal rate could reach 53.11% [3].

Due to the complexity and variety of microbial species in compost, externally added microbial agents often need to compete with indigenous microbes and quickly adapt to the special environment in the compost. Therefore, it is often unsustainable to add microbes to improve composting [24]. Cunningham et al. (2004) considered the immobilization of a microbial population including bacteria by using polyvinyl alcohol, which could provide a relatively favorable microenvironment for microorganisms as a buffer system such that it could better adapt to the micro-ecological environment and ensure the growth and reproduction of the microorganisms [25]. In addition, the immobilized carrier could act as an adsorbent for pollutants and effectively reduce them [26]. By immobilizing added microbial agents on biochar, the microorganisms might be protected by the carrier and avoid direct contact with the extreme environment within the compost, thereby increasing their survival rate [27,28]. The carrier material is an important factor that affects the activity and stability of the immobilized microorganism. The porosity and specific surface area of biochar are immense, and the pore diameter is generally less than 16 μm [29]. It is an inorganic carrier with a long use time, low price, good thermodynamic stability, and high mechanical strength. It is an ideal microbial immobilization carrier and has been widely used in the treatment of industrial wastewater and organic waste gas [30,31]. The immobilized-microorganism technique (IMT) could considerably enhance the removal of polycyclic aromatic hydrocarbons (PAHs) by using biochar as carriers [32]. It was found that cornstalk biochar had the best potential for NH_3_ emission reduction compared with the other types of biochar, such as those from bamboo, wood, laying hen manure, and coir [4].

Therefore, this study used cornstalk biochar as the carrier to compare the biochar load mixed bacteria method and to determine the effect of cornstalk biochar immobilized mixed bacteria on NH_3_ emissions. In addition, the mechanism of NH_3_ emission reduction was discussed through the detection of related physical, chemical, and microbial indices. The results of this study provide new concepts to preserve nitrogen and reduce NH_3_ during the process of manure composting.

## 2. Result

### 2.1. Effect of the Immobilized Mixed Bacteria of the Cornstalk Biochar Method on the NH_3_ Reduction of Composting

#### 2.1.1. NH_3_ Emission

**Figure 1:**
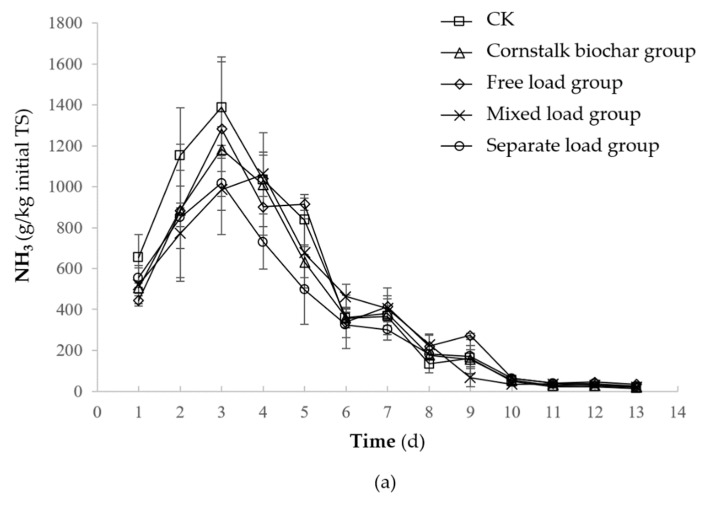

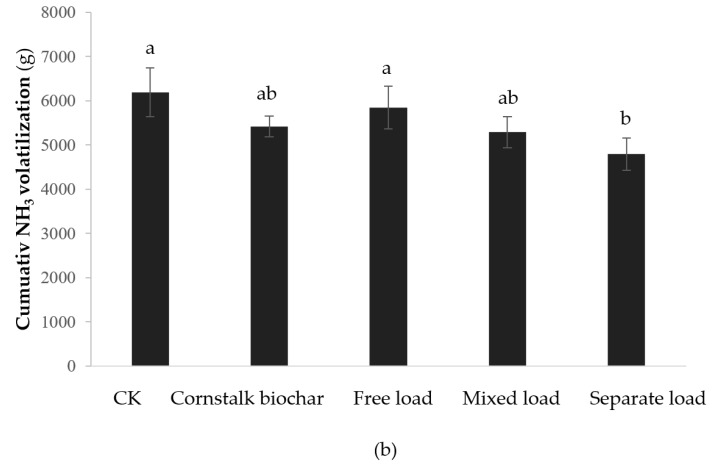
Changes of NH_3_ emissions in different load methods during composting; Note: (**a**) NH_3_ emission during the composting, (**b**) total NH_3_ emission (CK: as the control). Error bars represent the standard deviation (*n* = 3); TS: total solid; ^a–b^ Columns reporting different letters are significantly different (*p* < 0.05).

The NH_3_ emissions of all treatments first rose, subsequently declined, and finally tended to be stable (Figure 1a). The NH_3_ emission peak of the control, cornstalk biochar group, free load group, mixed load group, and separate load group appeared on the third, third, third, fourth, and third days, respectively. The NH_3_ emission peak value in the control, cornstalk biochar group, free load group, mixed load group, and separate load group was 1387.5 ± 247.8, 1183.7 ± 108.8, 1282.2 ± 329.5, 1060.5 ± 108.7, and 1017.0 ± 280.0 mg/kg, respectively. There was no significant difference between the peaks (*p >* 0.05). During the composting process, NH_3_ emissions in the separate load group were significantly lower than those in the control group (*p <* 0.05) while NH_3_ emissions in the other groups were not significantly different (*p >* 0.05).

The total NH_3_ emissions from the control, cornstalk biochar group, free load group, mixed load group, and separate load group were 6193.5 ± 548.8, 5423.6 ± 231.3, 5851.1 ± 482.9, 5291.0 ± 349.6, and 4792.9 ± 361.6 mg/day, respectively (Figure 1b). Compared with the control treatment, the total NH_3_ emissions of the cornstalk biochar group, free load group, mixed load group, and separate load group decreased by 12.43%, 5.53%, 14.57%, and 22.61%, respectively, although the separate load group was the only treatment that significantly reduced the release of NH_3_ (*p <* 0.05).

#### 2.1.2. Changes of Physicochemical Factors

**Figure 2:**
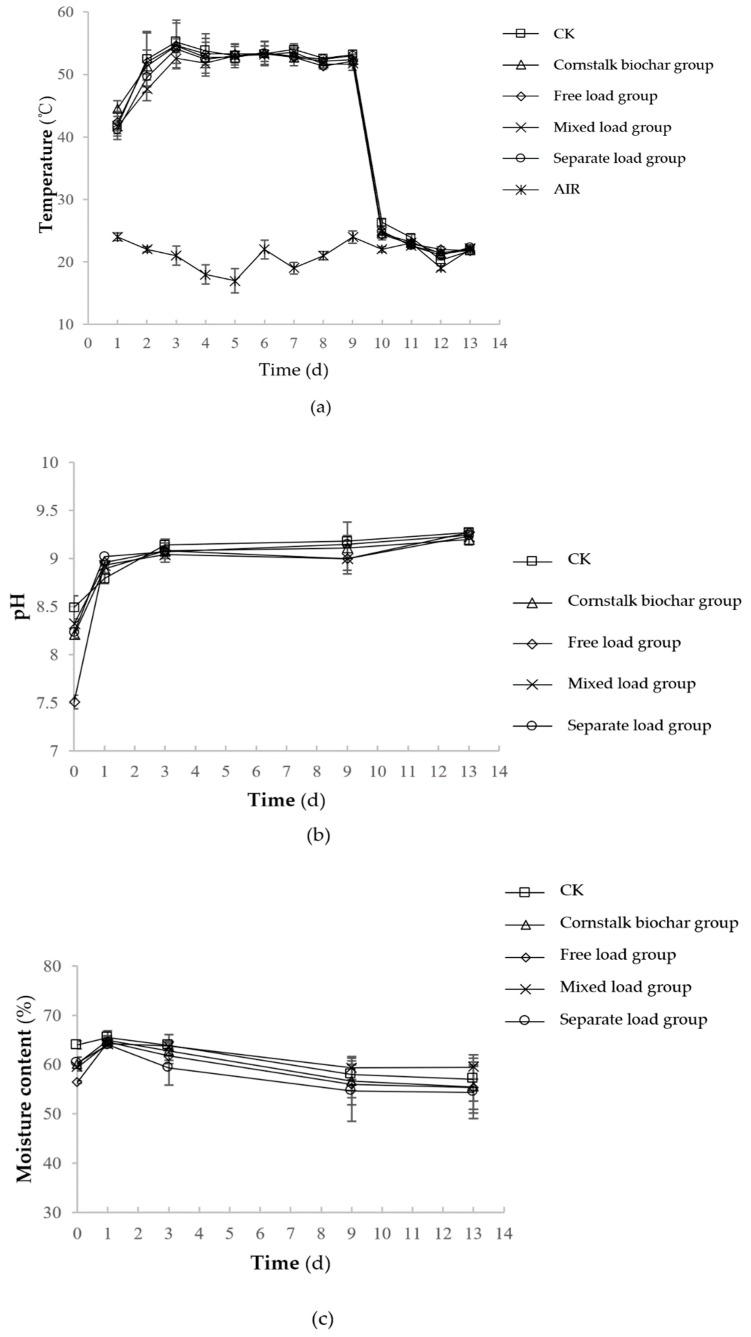
Evaluation of the temperature (**a**), pH (**b**) and moisture content (**c**) profile of different load methods during composting (CK: as the control); Note: Values are based on wet weight, and error bars represent the standard deviation (*n* = 3).

Stack temperature changes in each group are shown in Figure 2a. The trends of temperature in each group were similar, and all experienced three processes: A rising temperature period (0–3 days), a high temperature period (3–9 days), and a cooling and decomposing period (9–15 days). The reactor temperature exceeded 50 °C during the whole high temperature period, consistent with the “Sanitation Health Standards” (GB7959-87) requirements [33]. The highest composting temperatures in the control treatment, cornstalk biochar group, free load group, mixed load group, and separate load group were 55.2 ± 3.5, 54.6 ± 0.6, 54.7 ± 3.7, 53.6 ± 1.4, and 54.1 ± 1.5 °C, respectively, while the temperature of each group was not significantly different (*p >* 0.05).

The tendency of the pH to change in each treatment was the same (Figure 2b), with a rapid rise followed by gradual stability. During the composting process, the average pH values of the control treatment, cornstalk biochar group, free load group, mixed load group, and separate load group were 8.97 ± 0.29, 8.90 ± 0.36, 8.76 ± 0.64, 8.90 ± 0.31, and 8.94 ± 0.36, respectively, while the pH of each group was not significantly different (*p >* 0.05).

The moisture content in each group exhibited the same tendency (Figure 2c). The moisture content showed a slight increase and then gradually decreased, but it was generally stable. The initial moisture content of each treatment group was approximately 60%. The average moisture content of the control treatment, cornstalk biochar group, free load group, mixed load group, and separate load group was 61.7%, 60.0%, 58.8%, 61.3%, and 58.5%, respectively, during the whole composting period. At the end of the composting, the moisture content of the control treatment, cornstalk biochar group, free load group, mixed load group, and separate load group was 57.0 ± 4.3%, 55.5 ± 4.6%, 55.3 ± 5.2%, 59.5 ± 2.5%, and 54.3 ± 5.3%, respectively. There was no significant difference (*p >* 0.05) in the moisture content of each group during the whole composting process.

### 2.2. Simulated Composting Study on NH_3_ Emissions during Composting with Cornstalk Biochar Immobilized Mixed Bacteria

#### 2.2.1. Changes of Physicochemical Factors

**Figure 3:**
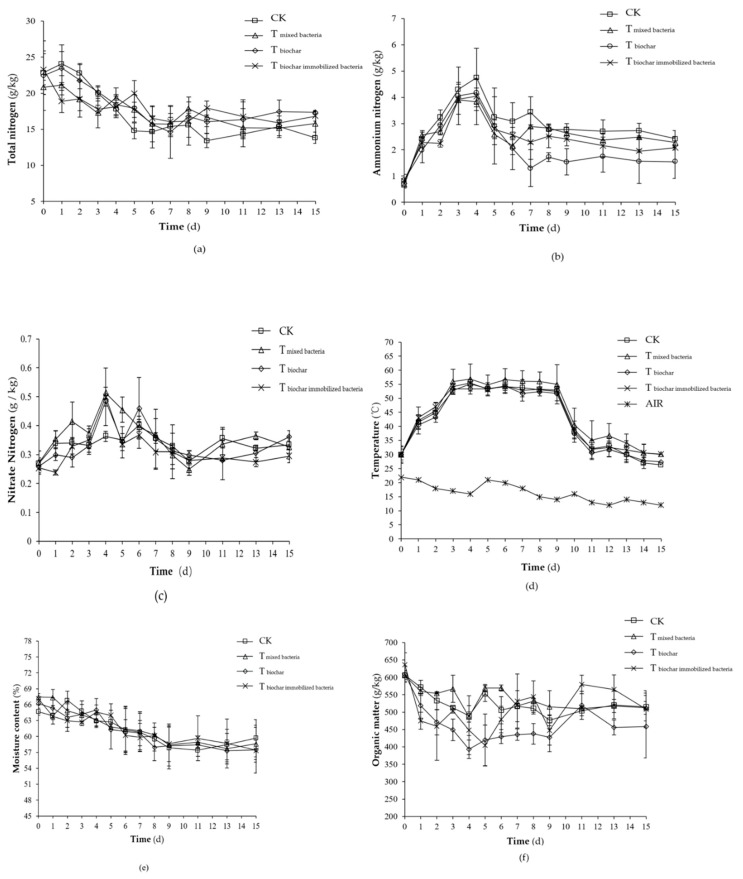

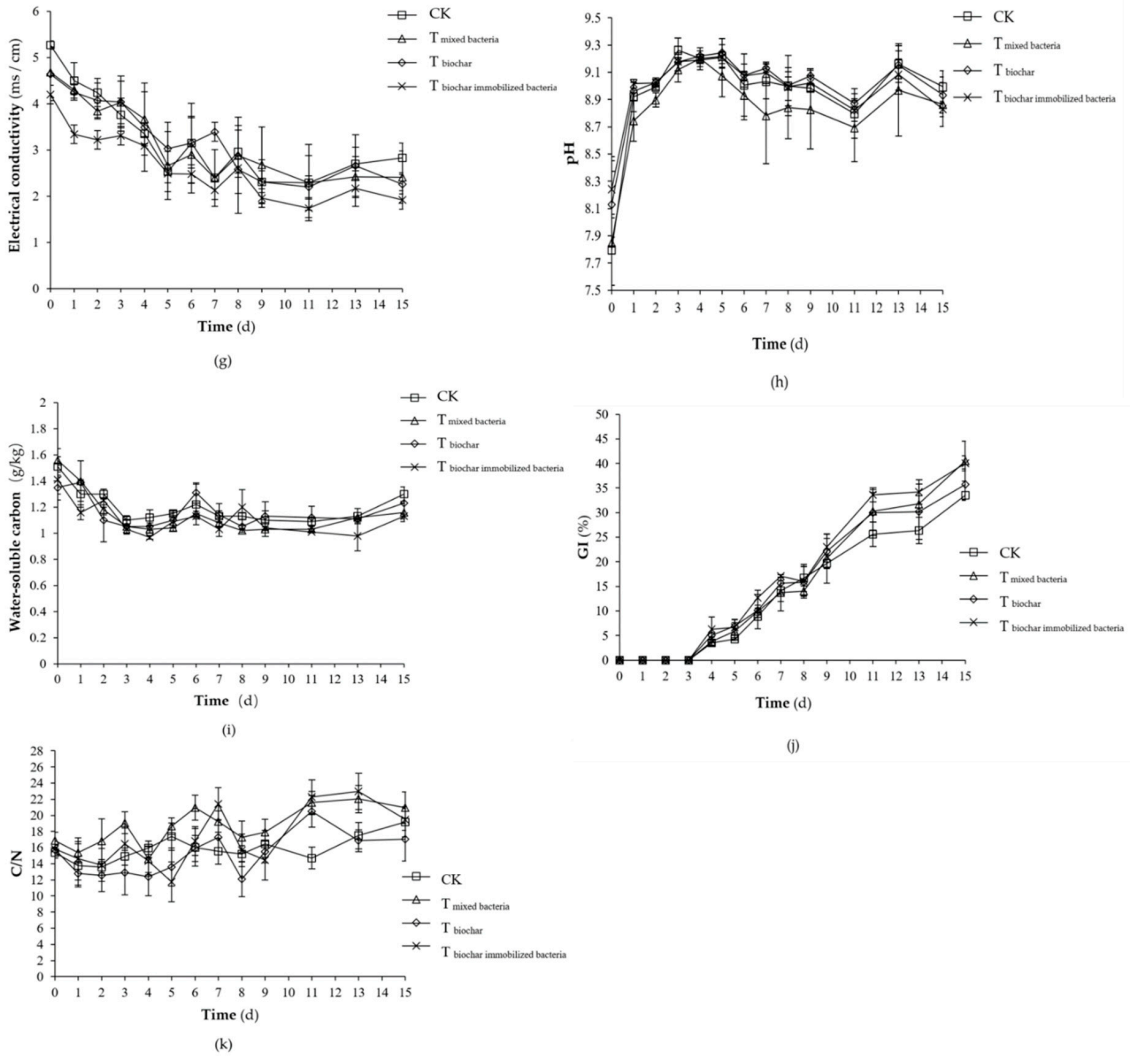
Evaluation of the total nitrogen (**a**), NH_4_^+^-N (**b**), NO_3_-N (**c**), temperature (**d**), moisture content (**e**), organic matter (**f**), electrical conductivity (**g**), pH (**h**), water-soluble carbon (**i**), seed germination rate (**j**), and C/N (**k**) profile of different treatments during composting (CK: as the control); Note: Values are based on wet weight and error bars represent the standard deviation (*n* = 3).

The total nitrogen change in each experimental group was largely the same, all of which were stable after initially decreasing (Figure 3a). During the first nine days in the period of temperature rise and high temperature, the NH_3_ aggressively volatilized, and the total nitrogen loss was faster due to the strong metabolic activity of the microorganisms. From the 10th day on, the nitrogen content slightly increased due to a lower amount of NH_3_ volatilization and less nitrogen loss due to the gradual decrease in temperature. During the whole composting process, the total nitrogen contents of each treatment were not different (*p >* 0.05). At the end of the composting process, the total nitrogen loss rates for the control treatment, T_mixed bacteria_, T_biochar_, and T_biochar immobilized bacteria_ were 39.5%, 24.2%, 22.7%, and 27.9%, respectively. Compared with the control treatment, the total nitrogen loss of the T_mixed bacteria_, T_biochar_ and T_biochar immobilized bacteria_ decreased by 38.7%, 42.6% and 29.4%, respectively, while the nitrogen loss could be significantly reduced by the T_mixed bacteria_, T_biochar_, and T_biochar immobilized bacteria_ (*p <* 0.05).

The ammonium nitrogen content of all the groups showed a trend of first increasing, subsequently decreasing, and finally maintaining a steady state (Figure 3b). In the early stage of composting, ammonium nitrogen increased because a large amount of amino acid was decomposed due to the massive degradation of nitrogen-containing organic mix reaching its peak around the fourth day. Next, the ammonium nitrogen began to rapidly transform into NH_3_ volatilization, and the ammonium nitrogen content decreased. After the temperature dropped, the nitrogen fixation of the microorganisms and the nitrification of the nitrobacteria also promoted the reduction of ammonium nitrogen. During composting, the peak values of ammonium nitrogen in the control treatment, T_mixed bacteria_, T_biochar_, and T_biochar immobilized bacteria_ were 4.75 ± 1.12, 3.90 ± 0.07, 4.17 ± 0.67, and 4.04 ± 0.31 g/kg, respectively. There was no significant difference in the peak value of ammonium nitrogen between treatments (*p >* 0.05). At the end of the composting, the ammonium nitrogen in the control treatment, T_mixed bacteria_, T_biochar_, and T_biochar immobilized bacteria_ was 2.42 ± 0.32, 2.28 ± 0.20, 1.54 ± 0.64, and 2.07 ± 0.07 g/kg, respectively. An analysis of the variance showed that during the whole composting process, the ammonium nitrogen content was as follows: T_biochar_ < T_mixed bacteria_ and T_biochar immobilized bacteria_ < control treatment (*p <* 0.05).

The nitrate nitrogen content in each group first increased and then decreased, and finally slightly increased again (Figure 3c). The initial temperature of the compost was relatively low, which was suitable for the growth and reproduction of the nitrifying bacteria and nitrification reaction, so the nitrate concentration increased. During the high temperature composting period, mesophilic nitrifying bacteria did not grow well due to the temperature exceeding 50 °C, which lead to decreased nitrification and a decreased nitrate nitrogen concentration. After entering the cooling maturity stage, the nitrifying bacteria grew again, and the nitrate nitrogen concentration increased again. At the end of the composting, the nitrate nitrogen contents of the control treatment, T_biochar_, T_mixed bacteria_, and T_biochar immobilized bacteria_ were 0.33 ± 0.05, 0.33 ± 0.02, 0.36 ± 0.00, and 0.30 ± 0.02 g/kg, respectively. During the whole composting process, the nitrate nitrogen content in the T_mixed bacteria_ was significantly higher than that in the other treatments (*p <* 0.05) while the other three treatments were not different (*p >* 0.05).

The trends of the temperature were similar to those observed in Section 2.1.2, where three temperature periods were identified: Rising temperature (0–3 days), high temperature (3–9 days), and cooling and decomposing (9–15 days) (Figure 3d). The highest composting temperature in the control treatment, T_mixed bacteria,_ T_biochar_, and T_biochar immobilized bacteria_ was 55.1 ± 1.3, 56.9 ± 4.3, 54.3 ± 1.0, and 55.4 ± 1.0 °C, respectively. During the composting process, the temperature of the T_mixed bacteria_ was significantly higher than that of the other treatments (*p* < 0.05), which were not different from each other (*p >* 0.05). It may be that the addition of composite microbial agent, microbial growth, and reproduction accelerated the increase in bio-heat, resulting in increased temperatures.

The moisture content in each group showed the same tendency, which was a gradual decrease followed by steady readings (Figure 3e). The average moisture content of the control treatment, T_mixed bacteria_, T_biochar_, and T_biochar immobilized bacteria_ was 61.6 ± 0.9%, 61.9 ± 0.4%, 61.3 ± 1.0%, and 61.5 ± 0.6%, respectively. At the end of the composting, the water content of the control treatment, T_mixed bacteria_, T_biochar_, and T_biochar immobilized bacteria_ was 59.7 ± 3.5%, 58.6 ± 3.1%, 57.5 ± 1.8%, and 57.4 ± 4.3%, respectively. There was no significant difference (*p >* 0.05) in the moisture content of each treatment during the whole composting process. The possible reason is that the moisture generated by the microbial decomposition of organic matter is difficult to be lost by evaporation in closed-reactor composting systems, resulting in no significant decrease in water content in the compost material [4,34].

The changes of the organic matter in the treatment groups were largely the same, showing a slight increase after the first decline and then maintaining a steady trend (Figure 3f). Organic matter decreased in the initial stage of the compost, which may be due to abundant nutrients, suitable temperature, microbial mass reproduction and metabolism, and the rapid degradation of organic matter. The organic matter content slightly increased late because of the “enrichment effect”. The initial organic matter of the control treatment, T_mixed bacteria_, T_biochar_, and T_biochar immobilized bacteria_ was 606.3 ± 12.1, 605.2 ± 15.8, 609.3 ± 23.7, and 636.1 ± 34.4 g/kg, respectively, although the initial organic matter in each treatment showed no significant differences (*p >* 0.05). At the end of composting, the organic matter of the control treatment, T_mixed bacteria_, T_biochar_, and T_biochar immobilized bacteria_ was 514.8 ± 39.6, 512.2 ± 18.2, 458.2 ± 89.7, and 508.7 ± 53.2 g/kg, respectively, and decreased by 15.1%, 15.4%, 24.8%, and 20.0% compared with the initial organic matter content, respectively. During the composting process, the organic matter loss of the T_biochar_ was significantly higher than that of the other treatments (*p <* 0.05), but no significant difference was found between the other treatments (*p >* 0.05).

Electrical conductivity reflects the total concentration of ions in the compost, which is a measure of whether the fertilizer is harmless. Electrical conductivity less than 4 ms/cm is generally considered as more desirable [35]. Each treatment in the compost gradually showed a steady trend after first decreasing (Figure 3g). At the end of the composting, the conductivity of the control treatment, T_mixed bacteria_, T_biochar_, and T_biochar immobilized bacteria_ was 2.83 ± 0.32, 2.41 ± 0.56, 2.26 ± 0.21, and 1.92 ± 0.32 ms/cm, respectively. The conductivity of the reactor with the T_biochar immobilized bacteria_ was significantly lower than that of the other treatments (*p <* 0.05), but no significant differences were found between the other treatments (*p >* 0.05). Compared with the other treatments, the total ion concentration in the T_biochar immobilized bacteria_ was lower, probably because the cornstalk biochar-loaded bacteria could adsorb some ions, resulting in lower conductivity.

The tendency of the pH to change in each treatment group was the same (Figure 3h). The pH rapidly rose, then slowly decreased, and gradually stabilized. During the 15-day composting process, the average pH values of the control treatment, T_mixed bacteria_, T_biochar_, and T_biochar immobilized bacteria_ were 8.95 ± 0.01, 8.83 ± 0.05, 9.02 ± 0.03, and 8.99 ± 0.02, respectively. During the whole composting process, the pH of the T_mixed bacteria_ was significantly lower than that of the other three treatments (*p* < 0.05) while there was no significant difference between the other three treatments (*p >* 0.05).

The trend of water-soluble carbon in each treatment group was the same (Figure 3i). All decreased during the first three days, increased slightly, and then decreased at the high temperature, and finally remained steady. At the beginning of the composting, the water-soluble carbon content in the control treatment, T_mixed bacteria_, T_biochar_, and T_biochar immobilized bacteria_ was 1.51 ± 0.08, 1.56 ± 0.09, 1.35 ± 0.10, and 1.41 ± 0.11 g/kg, respectively (*p >* 0.05). During the whole composting process, the water-soluble carbon content of the control treatment was significantly higher than that of the T_biochar immobilized bacteria_ (*p <* 0.05) but not significantly different from the T_mixed bacteria_ and T_biochar_ (*p >* 0.05).

The seed germination rate is an important index to determine the maturity and toxicity of the compost, and the direct influence of the fertilizer on the germination of the plant seeds can indicate whether the compost is harmful [14,36]. At the beginning of the composting, the germination rate of each group of seeds was zero (Figure 3j). At this time, the compost completely inhibited seed germination. From the third day onwards, the germination index of the seeds continuously increased, and there was still an upward trend at the end of the composting. By the 15th day, the seed germination rate of the control treatment, T_mixed bacteria_, T_biochar_, and T_biochar immobilized bacteria_ was 33.5 ± 0.8%, 40.5 ± 4.0%, 35.7 ± 3.2%, and 40.0 ± 1.5%, respectively. The seed germination rate was not satisfactory. However, considering that the actual compost production often takes a longer time and requires two fermentations, the measured water and fertilizer have great differences with the actual production, and the final germination rate of the test is still on the rise. It was expected that the seed germination rate would continue to rise. The seed germination rate at the end of the composting was the highest in T_biochar immobilized bacteria_ and T_mixed bacteria_, which was significantly higher than that in the control treatment (*p <* 0.05). During the whole composting process, the seed germination rates of the other three treatments were significantly higher than that of the control treatment (*p <* 0.05). The results showed that the addition of mixed bacteria, cornstalk biochar, and cornstalk biochar immobilized mixed bacteria can reduce the toxicity of compost products and promote compost maturity.

The trends of the solid phase C/N in each test group were basically the same, all of which were stable after an initial increase (Figure 3k). An analysis of variance showed that the solid C/N of the T_biochar_ was significantly lower than that of the other treatments (*p <* 0.05) during the composting process, which may be related to the high organic matter loss rate of the cornstalk biochar addition. At the end of the composting, the solid phase C/N of the control treatment, T_mixed bacteria_, T_biochar_, and T_biochar immobilized bacteria_ was 19.2 ± 1.9, 20.9 ± 1.9, 17.0 ± 2.7, and 19.5 ± 1.3, respectively. The relative initial C/N of each treatment increased with the exception of the biochar.

#### 2.2.2. NH_3_ Emission

**Figure 4:**
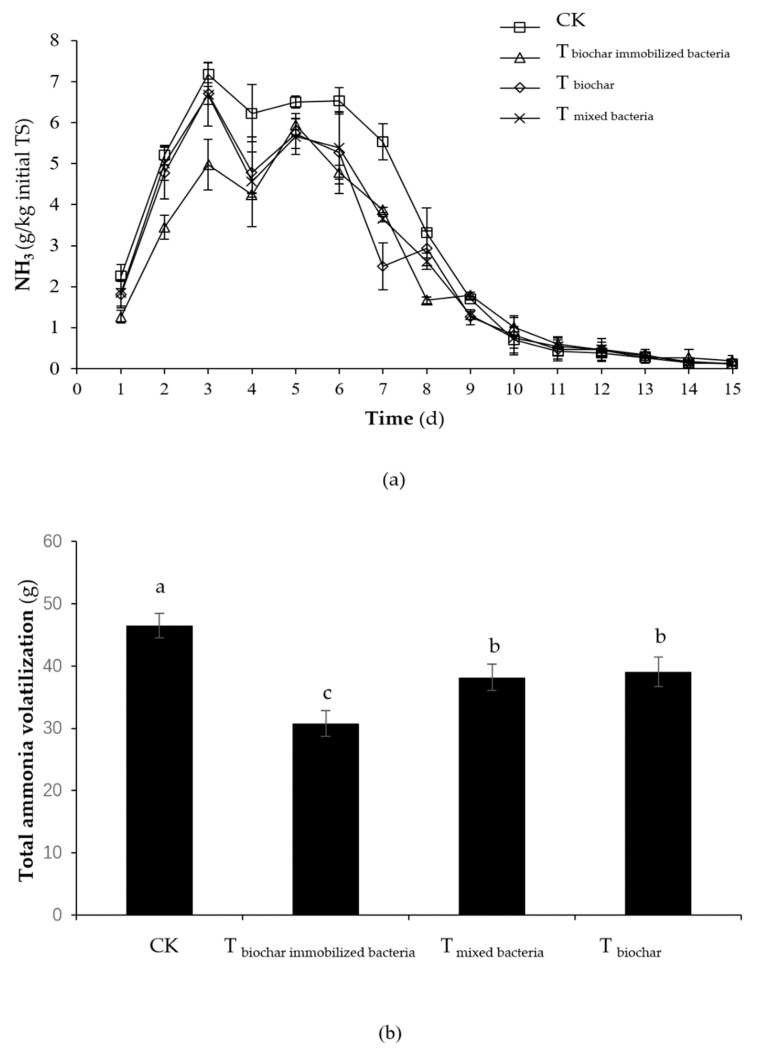
Changes in the NH_3_ emissions in different composting treatments; Note: (**a**) NH_3_ emission during the composting (**b**) total NH_3_ emission (CK: as the control). Error bars represent the standard deviation (*n* = 3); TS: total solid; ^a–c^ Columns reporting different letters are significantly different (*p* < 0.05).

The NH_3_ emissions of all the groups increased first, later declined, and finally tended to be stable (Figure 4a). The NH_3_ emission peak of the control treatment, T_biochar immobilized bacteria_, T_biochar_, and T_mixed bacteria_ appeared on the third, fifth, third, and third days, respectively. The peak delay of the T_biochar immobilized bacteria_ may be related to the immobilized-microorganism technique. The NH_3_ emission peak value in the control treatment, T_biochar immobilized bacteria_, T_biochar_, and T_mixed bacteria_ was 7.18 ± 0.29, 5.94 ± 0.28, 6.71 ± 0.27, and 6.68 ± 0.77 g/day, respectively. The NH_3_ emission peak in the T_biochar immobilized bacteria_ was significantly lower than that in the control treatment (*p <* 0.05). At 0–3 days of composting, the NH_3_ emission in the T_biochar immobilized bacteria_ was significantly lower than that in the other three groups (*p <* 0.05). In the composting period of 4 to 9 days, the NH_3_ emissions of the control treatment were significantly higher than those of the other three groups (*p <* 0.05). There was no significant difference (*p >* 0.05) in the NH_3_ emissions from 10 to 15 days of composting. This finding showed that compared with the control treatment, the addition of cornstalk biochar immobilized mixed bacteria can reduce NH_3_ emissions during composting and at high temperatures while the addition of cornstalk biochar and mixed microbial agents can reduce high-temperature NH_3_ emissions.

The total NH_3_ emissions from the control treatment, T_biochar immobilized bacteria_, T_mixed bacteria,_ and T_biochar_ were 46.5 ± 0.2, 30.8 ± 1.4, 38.1 ± 2.1, and 39.1 ± 2.0 g, respectively (Figure 4b). Compared with the control treatment, the T_biochar immobilized bacteria_, T_mixed bacteria_, and T_biochar_ significantly reduced the release of NH_3_ (*p <* 0.05). The total NH_3_ emissions of the T_biochar immobilized bacteria_, T_mixed bacteria,_ and T_biochar_ decreased by 33.8%, 18.0%, and 15.9% compared to the control treatment. In addition, the total NH_3_ emissions of T_biochar immobilized bacteria_ were significantly lower than those from T_mixed bacteria_ and T_biochar_ (*p <* 0.05), without any significant differences between T_mixed bacteria_ and T_biochar_ (*p >* 0.05).

#### 2.2.3. Total Number of Microorganisms

**Figure 5:**
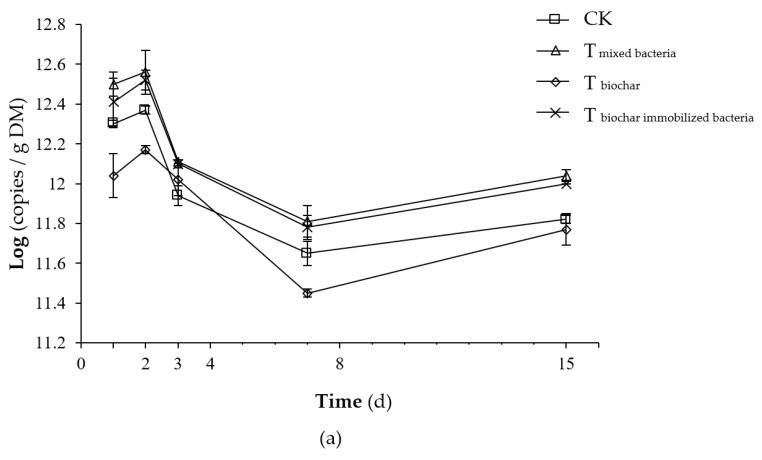

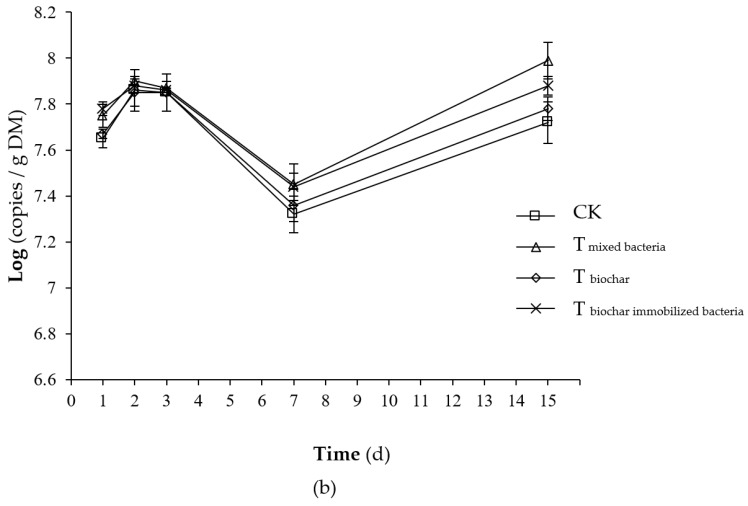
Total number of bacterial (**a**) and fungal (**b**) profiles for different composting treatments (CK: as the control); Note: Error bars represent the standard deviation (*n* = 3).

During the compost process, the total bacteria in each treatment tended to be the same, with all rising rapidly and later declining rapidly and finally increasing slightly (Figure 5a). Before the second day, the temperature rise was relatively slow, which is more suitable for bacterial growth and reproduction, so the total amount of bacteria increased slightly. By day 3, the composting started to enter the high temperature period. The thermophilic bacterial activity began to increase, but the majority of non-high temperature-resistant bacteria were inhibited, so that the total bacterial number decreased rapidly. After reaching the high temperature, the primary body of the mesophilic bacteria was restored and reproduced. Thus, the total bacterial population increased.

During the process of composting, the total bacterial copy number of each treatment was between 11.5 and 12.6. At the beginning of the composting, T_mixed bacteria_ and T_biochar immobilized bacteria_ were significantly higher than T_biochar_ (*p <* 0.05), but there was no significant difference between the control treatment and T_biochar_ (*p >* 0.05). During the composting process, the logarithm of the total bacterial number of T_mixed bacteria_ and T_biochar immobilized bacteria_ was significantly higher than that of the control treatment (*p <* 0.05), and that of the control treatment was significantly higher than that of T_biochar_ (*p <* 0.05). There was no significant difference between T_mixed bacteria_ and T_biochar immobilized bacteria_ (*p >* 0.05). This finding may be because T_mixed bacteria_ and T_biochar immobilized bacteria_ were all added with a bacterial agent, thereby increasing the total amount of bacteria.

The total number of fungi in each group changed in a consistent manner, all rising slightly and later decreasing, and finally increasing (Figure 5b). The temperature at the start of the composting was lower, allowing the fungi to multiply and increase their numbers. Fungal heat-resistant activity was inhibited in the high temperature period, and the number decreased. With a lower temperature at maturity, fungal activity was restored, and the total amount of fungi increased.

During the whole process of composting, the number of total fungi copies in each group was between 7.32 and 7.99. An analysis of variance showed that on the first day of composting, T_mixed bacteria_ and T_biochar immobilized bacteria_ were significantly higher than those in the control treatment (*p <* 0.05). During the whole composting process, the logarithm of the total fungal copy number of T_mixed bacteria_ and T_biochar immobilized bacteria_ was significantly higher than that of the control and T_biochar_ (*p <* 0.05). There was no significant difference between the control and T_biochar_ (*p >* 0.05). The possible reason is that T_mixed bacteria_ and T_biochar immobilized bacteria_ separately added the complex bacteria in different ways to increase the total amount of fungi.

#### 2.2.4. Number of Added Bacteria

**Figure 6:**
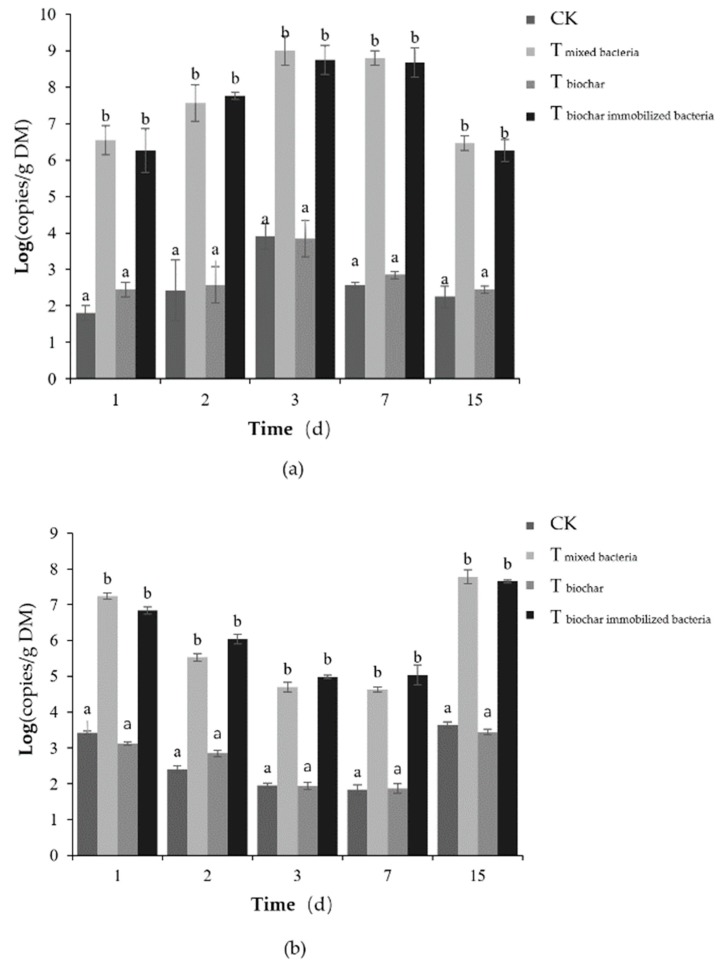

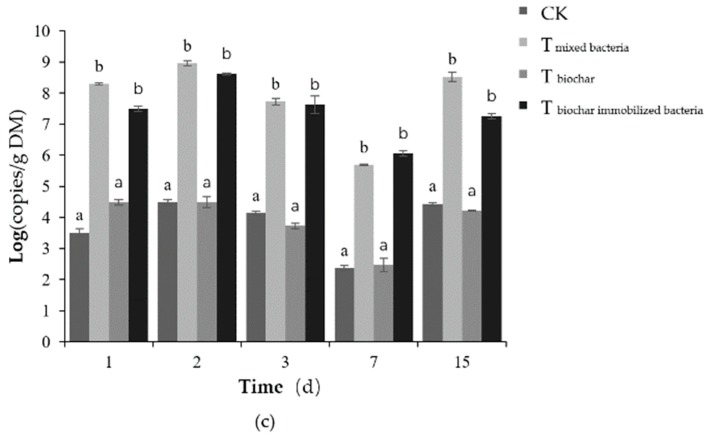
Changes in the number of *Bacillus stearothermophilus* (**a**), *Candida utilis* (**b**), and *Bacillus subtilis* (**c**) for different composting treatments (CK: as the control); Note: Error bars represent the standard deviation (*n* = 3); An analysis of variance in the figure is the same day, data comparison between the different composting treatments; ^a–b^ Columns reporting different letters are significantly different (*p* < 0.05).

The number of *Bacillus stearothermophilus* in each test group tended to be the same, all increasing and then decreasing (Figure 6a). Since *Bacillus stearothermophilus* is a thermophilic bacterium, its optimal growth temperature is 50–65 °C. As the temperature gradually increased, the number of *Bacillus stearothermophilus* gradually increased. The temperature decreased during the late composting phase, and *Bacillus stearothermophilus* gradually lost its activity, resulting in a decrease in its quantity. During the whole composting process, the logarithm of the number of *Bacillus stearothermophilus* in T_mixed bacteria_ and T_biochar immobilized bacteria_ was significantly higher than that of the control and T_biochar_ (*p* < 0.05). There was no significant difference between the control and T_biochar_ (*p* > 0.05). The logarithm of the number of *Bacillus stearothermophilus* in T_mixed bacteria_ and T_biochar immobilized bacteria_ was between 6.26 and 9.00 while the control and T_biochar_ were between 1.81 and 3.91. The possible reason is that T_mixed bacteria_ and T_biochar immobilized bacteria_ added *Bacillus stearothermophilus*.

The changes in the number of *Candida utilis* in each test group tended to be the same, all decreasing and then increasing (Figure 6b). In the early stages of the composting, the temperature gradually increased while the mesophilic bacterial *Candida utilis*’s growth and reproduction were inhibited. After the composting phase cooled down, *Candida utilis* resumed its activity from the spore state, therefore increasing its quantity. During the whole composting process, the logarithm of the number of *Candida utilis* in T_mixed bacteria_ and T_biochar immobilized bacteria_ was significantly higher than that of the control and T_biochar_ (*p <* 0.05). There was no significant difference between the control and T_biochar_ (*p >* 0.05). The logarithm of the copy number of *Candida utilis* in T_mixed bacteria_ and T_biochar immobilized bacteria_ was between 4.63 and 7.78 while that in the control and T_biochar_ was between 1.84 and 3.64. The possible reason could be that T_mixed bacteria_ and T_biochar immobilized bacteria_ all have mixed bacteria added.

The number of *Bacillus subtilis* in each test treatment tended to change consistently, all slightly increasing, then decreasing, and finally increasing (Figure 6c). At the beginning of the composting, the temperature rose slowly, and the mesophilic bacterial *Bacillus subtilis* therefore grew and bred in small quantities. After reaching the high temperature period, the growth of *Bacillus subtilis* was inhibited, and it produced spores. *Bacillus subtilis* resumed its activity from the sporulation state after entering the cooling maturity stage, increasing in number. During the whole composting process, the logarithm of the copy number of *Bacillus subtilis* in T_mixed bacteria_ and T_biochar immobilized bacteria_ was significantly higher than that of the control treatment and T_biochar_ (*p <* 0.05). There was no significant difference between the control treatment and T_biochar_ (*p >* 0.05). The logarithm of the number of *Bacillus subtilis* in T_mixed bacteria_ and T_biochar immobilized bacteria_ was 5.69–8.96 while that in the control treatment and T_biochar_ was between 2.37 and 4.49. The probable cause is that the complex bacteria added by T_mixed bacteria_ and T_biochar immobilized bacteria_ contained a large amount of *Bacillus subtilis*.

#### 2.2.5. Composition of Bacterial Communities

The abundance information of each OTU (operational taxonomic units) of each sample was counted. The abundance of OTU initially indicates the species richness of the sample. A total of 37,290 OTUs were generated from 60 samples.

Alpha Diversity is a reflection of sample diversity, including PD_whole_tree, Chao1, observed species, and Shannon four indices. As seen from the Chao1 exponential graph in Figure S1b (Supplementary Material), all the samples averaged more than 200 based on the total number of sequences read by the OTU. The curve in the Shannon index graph tended to be flat, indicating that the sequencing depth covered all the species in the sample (Figure S1d, Supplementary Material). It can be seen from the descriptive graphs of all the indices that the trend of the species diversity in all of the samples was consistent.

The composting process had a more pronounced effect on the OTU abundance of the samples compared to the different treatments (Figure S2, Supplementary Material). On the first day of composting, the clusters in each treatment were close. From the second day, the OTU abundance clustering distance gradually increased in each treatment. By the seventh day, the OTU abundance clustering distance of each treatment reached its maximum. By the 15th day, the OTU abundance clustering of each treatment was closer again. The results indicated that the bacterial colonies were similar at the beginning and the end of the compost while the colony structures were relatively different during the heating phase and the high temperature phase.

Compared to the other treatments, except for the 15th day, the samples of T_mixed bacteria_ clustered closer, and the bacterial community structure was more similar. Compared with the control and T_biochar_, T_biochar immobilized bacteria_ was relatively clustered. The microbial community structure of T_biochar_ was similar to that of the control treatment.

#### 2.2.6. Systematic Taxonomy Analysis

**Figure 7:**
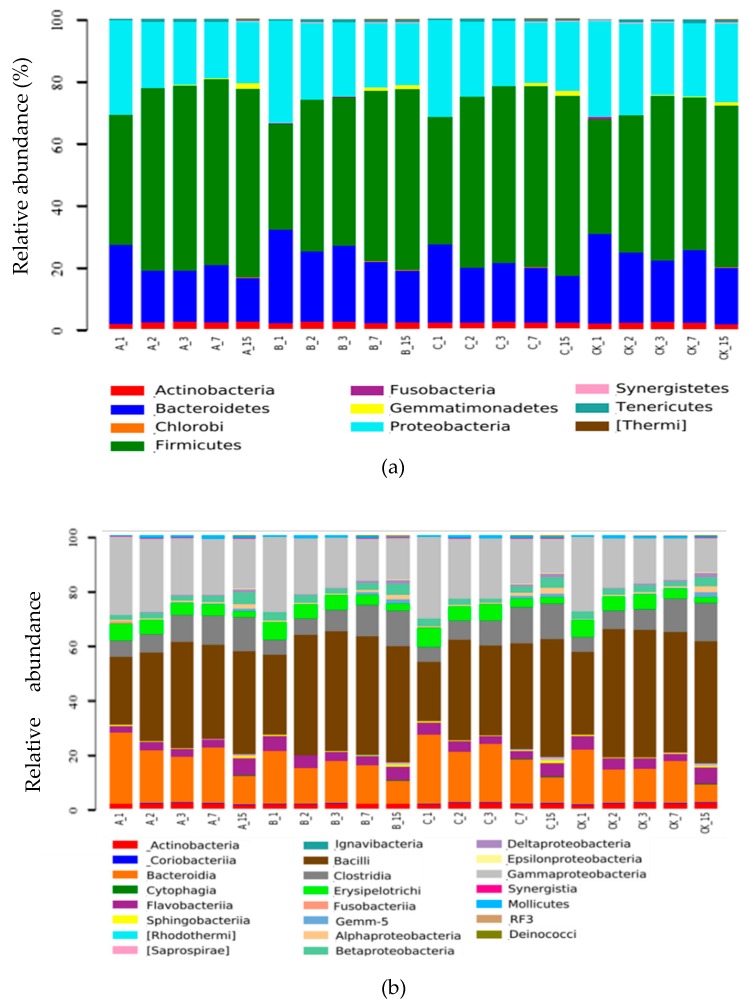

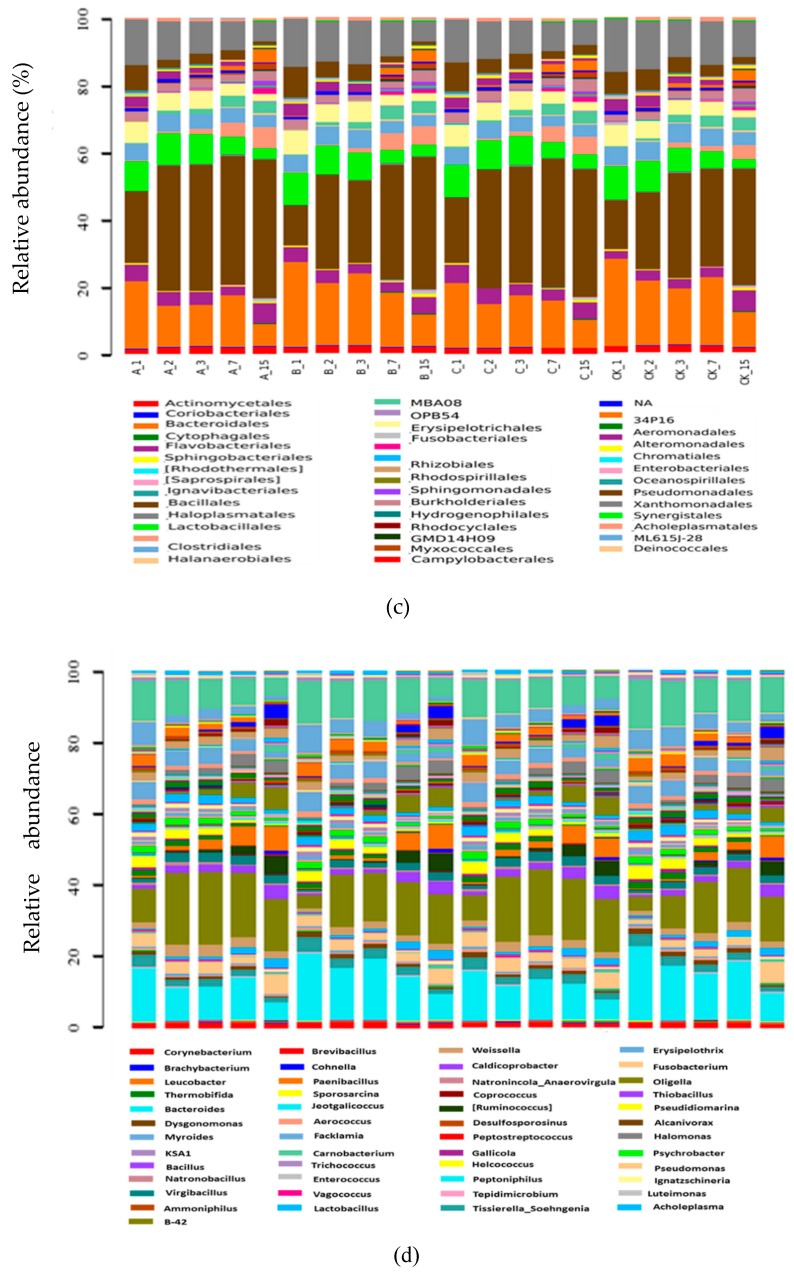
Variation of the basic information of the bacterial communities with different treatments; Note: (**a**) The relative abundance of the dominant bacterial taxonomic groups at the phyla level (related abundances exceed 1%) separated by 16S rDNA gene sequences; (**b**) The relative abundance of the dominant bacterial taxonomic groups at the class level (related abundances exceed 1%) separated by 16S rDNA gene sequences; (**c**) The relative abundance of the dominant bacterial taxonomic groups at the order level (related abundances exceed 1%) separated by 16S rDNA gene sequences; (**d**) The relative abundance of the dominant bacterial taxonomic groups at the genera level (related abundances exceed 1%). CK, A, B, and C were the control treatment, T_mixed bacteria_, T_biochar_, and T_biochar immobilized bacteria_, respectively. 1, 2, 3, 7, and 15 represent compost samples on days 1, 2, 3, 7, and 15, respectively.

The dominant bacterial phyla (relative abundance over 1%) were demonstrated to be *Firmicutes*, *Proteobacteria*, and *Bacteroidetes*; the abundances were 34.1%–60.7%, 18.2%–32.9%, and 14.3%–30.1%, respectively (Figure 7a). With the progression of the compost, the relative contents of *Firmicutes* in each treatment increased gradually, and the relative abundance increased from the initial 34.1%–41.9% to 52.1%–60.7%. The primary reason for this finding was that after the start of the composting, the microbial metabolic activity was abundant, and the reactor temperature rose rapidly. The thick-walled microbial cell wall thickness (10–50 nm) is better adapted to the high-temperature environment [37], meaning that *Firmicutes* gradually became the dominant bacteria. When the compost temperature became too high, the relative content of *Proteobacteria* and *Bacteroidetes* gradually decreased. The relative abundance decreased from 30.3%–32.9% and 25.3%–30.1% at the beginning of the composting to 19.8%–25.6% and 14.3%–18.3% at the end of the composting, respectively. On the first day of composting, the content of the bacteria in T_mixed bacteria_ and T_biochar immobilized bacteria_ was significantly higher than that in the control treatment and T_biochar_ (*p <* 0.05). This is also true for the entire composting process (*p <* 0.05). This may be because two of the strains of bacteria added to the two groups belong to the *Firmicutes*.

The dominant bacterial classes were demonstrated to be *Bacilli*, *Gammaproteobacteria*, and *Bacteroidia* with abundances of 21.7%–47.0%, 12.5%–29.8%, and 6.6%–26.0%, respectively (Figure 7b). With the progression of the composting, the *Bacilli* in all of the treatments showed an increasing trend in composting, gradually becoming the predominant flora, while the content of *Gammaproteobacteria* and *Bacteroidia* gradually decreased. From the third day of composting, the content of *Betaproteobacteria* in T_mixed bacteria_ was significantly higher than that of the control and T_biochar_ (*p <* 0.05). During the whole composting process, there was no significant difference in the content of the *Gammaproteobacteria* between treatments (*p >* 0.05).

The dominant bacterial orders were demonstrated to be *Bacillales*, *Bacteroidales*, and *Xanthomonadales (*Figure 7c). With the progression of the compost, *Bacillales* in each treatment increased during composting while the number of *Bacteroidetes* and *Xanthomonas* gradually decreased. The possible reason was that *Bacillales* could produce spores, and therefore were more tolerant to high temperatures. On the first day of composting, the average relative abundance of *Bacillales* in T_mixed bacteria_ and T_biochar immobilized bacteria_ with the mixed bacteria was significantly higher than that in the control treatment and T_biochar_, and this persisted during the whole composting process (*p <* 0.05). This could be because two bacteria of the added complex bacteria belonged to *Bacillales*, and these two bacteria can promote the growth and reproduction of other *Bacillales*.

*Dysgonomonas* represented around 10% of the relative bacteria content during the composting (Figure 7d). The average content of *Lactobacillus* in the control group, T_mixed bacteria_, T_biochar,_ and T_biochar immobilized bacteria_ was 1.64%, 2.06%, 1.70%, and 1.94%, respectively. The results showed that adding compound bacteria could promote the growth of *Lactobacillus* in the composting process. In the composting process, the average relative content of *Bacillus* in each group was 1.70%, 2.28%, 1.80%, and 2.36%, respectively, which may be related to the presence of *Bacillus* in the added bacteria.

## 3. Discussion

This study shows that the addition of cornstalk biochar immobilized mixed bacteria can significantly reduce the release of NH_3_ (*p <* 0.05). On the one hand, the compound bacteria that are loaded play a role. There are two possible mechanisms by which these bacteria reduce NH_3_ release. First, the release of NH_3_ is strongly affected by temperature and pH [38,39]. The research in this paper shows that 1.5% of the composite microbial agent at the beginning of composting can affect the microbial community structure at elevated and high temperatures during the composting process (Figures S1 and S2). Meanwhile, this can significantly increase the relative abundance of lactic acid bacteria and *Bacillus stearothermophilus* (*p <* 0.05) and decrease the pH during the composting progress (*p <* 0.05). It has been reported that both lactic acid bacteria and *Bacillus stearothermophilus* produce acid during composting [40,41], therefore reducing the pH during the composting progress. Xie et al. (2012) added ammonia-oxidizing archaea to chicken manure compost and found that the addition of the ammonia-oxidizing archaea can significantly reduce the pH during composting, and therefore, the release of NH_3_ [42]. Second, the form of nitrogen also has a greater impact on the release of NH_3_. Nitrogen in the reactor is primarily in the form of ammonium nitrogen, nitrate nitrogen, organic nitrogen, and ammonia gas, in which ammonium nitrogen is easily converted to ammonia gas at higher pH and temperatures [43]. Since the total amount of nitrogen in the compost body is fixed, if the content of ammonium nitrogen that is easily converted into ammonia gas is reduced, and the content of relatively more stable organic nitrogen and nitrate nitrogen is increased, the nitrogen can be effectively reduced to release NH_3_ [44]. Research has shown that there is a significant positive correlation between the ammonium nitrogen content and NH_3_ release during compost [45]. Kuroda et al. (2004), adding *Bacillus* into composting, which was isolated from compost made of animal wastes, found that both of the bacteria promoted the transformation of water-soluble ammonium nitrogen, the formation of water-soluble organic nitrogen, and participated in the preservation of nitrogen [46]. This study shows that T_mixed bacteria_ can increase the relative abundance of β-proteobacteria and *Candida utilis* (*p <* 0.05), decrease the ammonium nitrogen and γ-proteobacteria content (*p <* 0.05), and increase the accumulation of nitrate nitrogen (*p <* 0.05). Studies indicate that β-proteobacteria are the primary nitrifying bacteria in compost microbes, which can promote the conversion of ammonium nitrogen to nitrate nitrogen [47,48,49], and γ-proteobacteria are the denitrifying bacteria [50,51,52]. *Candida utilis* can synthesize single-cell proteins using ammonium sources [53,54], nitrates, and urea as nitrogen sources [55]. By storing nitrogen in a relatively stable form, the volatilization of ammonia gas can be suppressed.

On the other hand, the properties of biochar were utilized, which are a large specific surface area and a rich porous structure. The results of our study demonstrated that the total NH_3_ production was significantly decreased for the treatments with biochar compared to the control treatment (*p <* 0.05). Chen et al. (2017) found that adding 10% cornstalk biochar to chicken manure compost could reduce the total NH_3_ emissions by 24.8% [4]. The above is probably related to the lower NH_4_^+^-N concentration. In this experiment, the ammonium nitrogen content of T_biochar_ was significantly lower than that of the other groups (*p <* 0.05). Chowdhury et al. (2014) showed that biochar can adsorb NH_4_^+^-N in chicken manure straw compost and reduce the accumulated NH_3_ emissions by 11%–21% [56]. Secondly, biochar has a large specific surface area (68.25 m^2^/g) and a rich porous structure (Table S1). It can directly adsorb the NH_3_ produced in compost and reduce NH_3_ emissions [57,58]. Saito et al. (2000) and Lehmann et al. (2007) showed that the specific surface area and pore volume of biochar determines its physical adsorption capacity [59,60]. The larger the specific surface area, the larger the adsorption capacity and the better the adsorption effect.

In actual production, the compost primarily uses large-scale two-time fermentation, which is different from the small-scale one-time fermentation employed in this study. Biologic immobilized technology provides protection to microorganisms through the use of carriers, thereby reducing the impact of the environment on them, and enhancing their activity and stability [25,28]. Therefore, it is necessary to verify the results of this experiment in actual production, which deserves further study.

## 4. Materials and Methods

### 4.1. Composting Materials

The composting materials comprised a mixture of laying hen manure, sawdust (2 mm mesh size), straw biochar (0.42 cm^3^/g pore size), and mixed bacteria, which were used as compost amendments. The mixed bacteria consisted of *Bacillus stearothermophilus*, *Candida utilis*, and *Bacillus subtilis* in a 1:2:1 ratio, which were purchased from the Center of Microbial Culture Preservation in Guangdong. The strains and their ratios were chosen on the basis of their maximum effectiveness in the removal of NH_3_, and antagonistic interactions between them [3]. Laying hen manure was collected from a chicken farm located at the Institute of Animal Science, South China Agricultural University, while sawdust residue was from the Zengcheng District, Guangzhou, China. The purpose of using sawdust (SW) was to adjust the C/N ratio and water content of the composting pile. Straw biochar was purchased from the Yaoshi Charcoal-Production Company located in Hangzhou. The straw biochar was pyrolyzed at 450–500 °C. The biochar was sieved through a 0.2-cm screen before being immobilized with mixed bacteria [61]. The main physicochemical characteristics of the raw materials and biochar are shown in Table S1 (Supplementary Material).

### 4.2. Experimental Design

#### 4.2.1. Immobilized Mixed Bacteria of the Cornstalk Biochar Method

Twenty-five 2.5-L airtight laboratory containers were used for this compost research. Each container was filled with fresh laying hen manure (498.3 g), sawdust (124.7 g, which is 25% of the manure wet weight), and different load methods of cornstalk biochar immobilized composite bacteria. The experiment was divided into four treatments: Cornstalk biochar (added cornstalk biochar), free load (added mixed bacteria and biochar separately), mixed load (added biochar with mixed bacteria immobilized in it), and separate load (each bacterium applied over biochar independently). The basic mixture of manure and sawdust (without the biochar) was used as a control (CK) in this study. Each treatment was repeated five times. The moisture content of the compost mixture was adjusted to an initial water content of 60%–70% (*w*/*w*, wet weight). The moisture content was no longer adjusted throughout the composting period. The stack was aerated intermittently with an electromagnetic air pump (ACO-009D, HAILEA, Chaozhou, China) and ventilated at a wind speed of 0.20 m^3^/h for 15 min per hour to provide oxygen through the compost. A thermometer was used to monitor the temperature in the composting process in real time.

#### 4.2.2. Simulated Composting Study on NH_3_ Emissions during Composting with Cornstalk Biochar Immobilized Mixed Bacteria

Twenty 19-L airtight laboratory containers were used for the compost research [4]. Each container was filled with fresh laying hen manure (6.2 kg), sawdust (1.3 kg, which is 21% of the manure wet weight), and different types of exogenous additives. The treatments were labeled T_mixed bacteria_ (1.5% mixed bacteria added), T_biochar_ (10% cornstalk biochar added), and T_biochar immobilized bacteria_ (10% biochar added with 1.5% of complex bacteria). Our previous study used 1.5% and obtained a better ammonia mitigation rate. The basic mixture of the manure and sawdust (without biochar) was used as a control (CK) in this study. Each treatment was repeated five times. The moisture content of the compost mixture was adjusted to an initial water content of 60%–70% (*w*/*w*, wet weight). The moisture content was no longer adjusted throughout the composting period. The stack was aerated intermittently with an electromagnetic air pump and ventilated at a wind speed of 0.20 m^3^/h for 15 min per hour to provide oxygen through the compost. A PT100 temperature probe provided real-time monitoring of the temperature during the composting process.

### 4.3. Sample Collection

#### 4.3.1. Immobilized Mixed Bacteria of the Cornstalk Biochar Method 

Composting and gas samples were collected on days 0, 1, 3, 8, and 12. Each stack was blended manually three minutes prior to daily sampling [62]. Ten-gram samples of the compost were collected. Each closed container was also equipped with a suction pump and the gas generated by the reactor progressed into the sulfuric acid absorption bottle for NH_3_ collection.

#### 4.3.2. Simulated Composting Study on NH_3_ Emissions during Composting with Cornstalk Biochar Immobilized Mixed Bacteria

Composting and gas samples were collected on days 0, 1, 2, 3, 4, 5, 6, 7, 8, 9, 11, 13, and 15. Each container had an automatic rotation device that homogenized the material, and each stack was rotated three minutes prior to daily sampling [62]. Twenty-gram samples of the compost, medium, and lower layer were collected for a total of 60 g. The compost was collected on the middle and lower 20 g samples for a total of 60 g. After mixing, 5 g were sampled and preserved at −80 °C to determine the microbial indicators. Fifty-five grams were sampled and fixed with 0.45 M sulfuric acid at 4 °C to determine the physical and chemical indicators. Each closed container was also equipped with a suction pump and the gas generated by the reactor progressed into the sulfuric acid absorption bottle for NH_3_ collection.

### 4.4. Bacteria Loading Rate Determination 

Twenty grams of *Bacillus stearothermophilus*, *Candida utilis, Bacillus subtilis*, and complex bacteria, respectively, were added to a 1000-mL conical flask filled with 200 mL of LB liquid medium. After oscillating at 160 r/min for 30 min, 5 mL of supernatant was sampled for serial dilution plating to determine the bacterial content. After adding 40 g of biochar to the flask, the operation was repeated. The loaded biochar was removed, and it was sealed at 4 °C for later use.

By calculating the bacterial content before and after the load, the loading efficiency of the microbial agent was determined and enabled the examination of whether the biochar was a good carrier for the microbial agent. The bacterial load rate formula is as follows:

Bacterial loading rate = (pre-loading bacterial content-bacterial content after loading)/(pre-loading bacterial content) × 100%

As seen from Table S2 (Supplementary Material), the bacterial load rate results ranged from 95.07% to 97.35%, and the bacterial load averaged 96.17%, which could ensure the complete load of the complex bacteria. 

### 4.5. Analytical Methods

#### 4.5.1. Gaseous Measurements

The NH_3_ in the sulfuric acid solution was determined by a Nessler′s Reagents spectrophotometer (uv1800, AUCY, Shanghai, China) [4].

#### 4.5.2. Physicochemical Analyses

The conductivity (EC) was measured using a conductivity meter (DDS-307, LEICHI, Shanghai, China), and a pH meter (PB-10, Sartorius AG, Göttingen, Germany) was used to measure the pH of water-soluble extracts of 1:10 (*w*/*v*, dry weight basis) [63]. The moisture content was determined by drying at 105 °C to a constant weight [64]. The water-soluble carbon content was determined using the water extraction-potassium dichromate oxidation-volumetric method. After centrifugation, the supernatant of the composting samples was used to cultivate the seeds of cress. The control treatment was cultured with distilled water. The number and root length of the germinating seeds were calculated, and the seed germination index (GI) was derived from the following formula [14]: GI (%) = (the seed germination rate of the compost treatment group)/(the seed germination rate of the control treatment was 100.) The ammonium nitrogen and the nitrate nitrogen were determined by potassium chloride-distillation titration [63]. The total nitrogen was determined by the modified micro-Kjeldahl digestion method with an automatic Kjeldahl apparatus (KDY-9830, Tongrunyuan, Beijing, China) [65]. The muffle furnace at 550 °C for 6 h was used to determine the organic matter content.

#### 4.5.3. Total DNA Extraction and PCR

The microbial total DNA of the composting samples was extracted using an E.Z.N.A.^TM^ Soil DNA Kit (Omega, Norcross, Georgia, USA). The compost sample’s total microbial DNA was subjected to an ordinary PCR reaction using specific primers. The primer sequences and PCR reaction programs used are shown in Tables S3 and S4 (Supplementary Material). 

#### 4.5.4. Detection of Bacterial and Fungal Diversity

##### Total DNA Extraction and PCR Amplification

An E.Z.N.A.^TM^ Soil DNA Kit was used to extract the total microbial DNA of the compost samples. The V3–V4 region of the bacteria 16S ribosomal DNA gene was amplified by the polymerase chain reaction (PCR) (95 °C for 5 min, followed by 25 cycles at 95 °C for 30 s, 56 °C for 30 s, and 72 °C for 40 s, and a final extension at 72 °C for 10 min) using the primers F (5′-ACTCCTACGGGAGGCAGCAG-3′) and R (5′-GGACTACHVGGGTWTCTAAT-3′). The ITS1 region of the fungal 18S ribosomal DNA gene was amplified by the polymerase chain reaction (PCR) (94 °C for 5 min, followed by 30 cycles at 94 °C for 30 s, 55 °C for 30 s, and 72 °C for 30 s, and a final extension at 72 °C for 10 min) using the primers F (5′-CTTGGTCATTTAGAGGAAGTAA-3′) and R (5′-TGCGTTCTTCATCGATGC-3′).

##### Processing of Sequencing Data

The DNA sequence analysis used Illumina MiSeq paired-end sequencing. Raw data generated from the high-throughput sequencing run was processed and analyzed following the pipelines of the QIIME platform [66]. The sequence reads were trimmed such that the average quality score for each read was above 20. After the sequences were joined, sequences less than 100 bp in length were removed using Mothur (filtering minlength = 100, maxhomop = 10, maxambig = 0). QIIME was used to remove chimeras and error sequences. Sequence clustering was performed using Uclust (QIIME) with a similarity cutoff of 97%, after which the samples were clustered into operational taxonomic units (OTUs). The abundance information of each sample OTU was counted. The alpha-diversity (Shannon index, Chao1 index, phylogenetic diversity, and observed number of OTUs) was calculated to access the bacterial diversity and richness using the QIIME software. The rarefaction curves and bacterial community distribution bar chart were generated using R software (v3.1.1).

### 4.6. Data Analysis

The general linear model (GLM) of SPSS19.0 was used to perform a one-way analysis of variance (ANOVA) on the data obtained by group design. Multiple comparisons used the Tukey test. A difference of *p* < 0.05 was considered to be statistically significant. The statistical results are expressed as the mean ± standard error (M ± SE).

## 5. Conclusions

This is the first study on the cornstalk biochar load mixed bacteria method that has demonstrated the effect and mechanism of action of cornstalk biochar immobilized mixed bacteria on NH_3_ emissions during composting. The results found suggest that efficient composting systems with a stable microbial agent reduce NH_3_ emissions, and characterize the relationship between NH_3_ emissions and the microbial community and physicochemical parameters in the composting process. The results showed that the separate load bacteria were the better cornstalk biochar immobilized mixed bacteria method and significantly reduced NH_3_ emissions compared with the other treatments (*p <* 0.05). This might be due to the adsorption of cornstalk biochar and the increase of the relative abundance of lactic acid bacteria. These results are important for the provision of new concepts to preserve nitrogen and reduce NH_3_ during the process of manure composting. Further investigation is still necessary to determine the effect of the cornstalk biochar load mixed bacteria method during large-scale composting in different areas.

## Supplementary Materials

The following are available online. Figure S1: Alpha dilution curve of the sample, Figure S2: Sample cluster analysis, Table S1: Primary physicochemical characteristics of the raw materials (dry weight basis): laying hen manure, sawdust and biochar before composting, Table S2: Bacterial loading rate, Table S3: The specific primers for bacteria, fungi, Bacillus stearothermophilus, Candida utilis, Bacillus subtilis, Table S4: The ordinary PCR reaction program for bacteria, fungi, Bacillus stearothermophilus, Candida utilis, Bacillus subtilis.
