# Supplementary material for: Effect of Cornstalk Biochar Immobilized Bacteria on Ammonia Reduction in Laying Hen Manure Composting
Source: Molecules. 2020 Mar 28;25(7):1560. doi: 10.3390/molecules25071560 (PMC7181132; doi:10.3390/molecules25071560)
Supplement: Supplementary file 1 [file molecules-25-01560-s001.pdf]

molecules

MDPI

Article

Effect of Cornstalk Biochar Immobilized Bacteria on Ammonia Reduction in Laying Hen Manure Composting

Huaidan Zhang [1], Jeremy N. Marchant-Forde [2], Xinyi Zhang [1] and Yan Wang [1,2,3,*]

[1] College of Animal Science, South China Agricultural University, Guangzhou 510642, China; zhd520888@163.com (H.Z.); zsz@stu.scau.edu.cn (X.Z.)

[2] Livestock Behavior Research Unit, USDA-ARS, West Lafayette, IN 47907, USA; jeremy.marchant-forde@usda.gov

[3] Guangdong Provincial Key Lab of Agro-Animal Genomics and Molecular Breeding and Key lab of Chicken Genetics, Breeding and reproduction, Ministry of Agriculture, Guangzhou 510642, China.

* Correspondence: ywang@scau.edu.cn; Tel.: +86-20-85280279; Fax: +86-20-85280740

Received: 20 February 2020; Accepted: date; Published: 28 March 2020

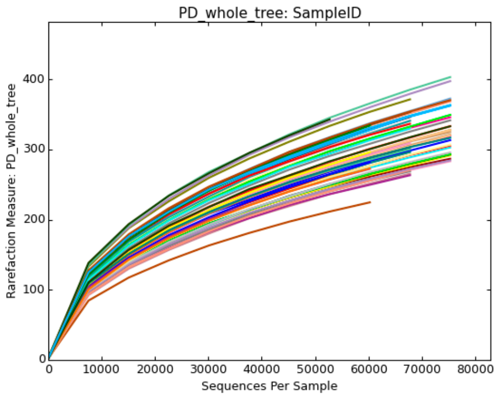

(a)

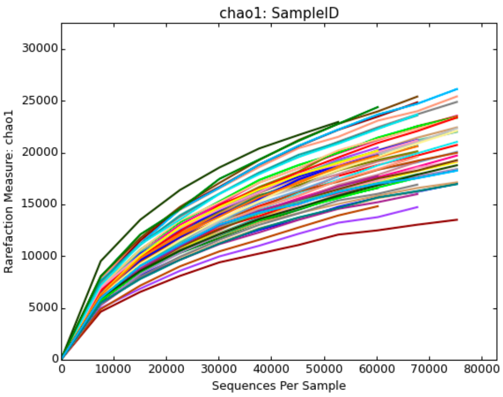

(b)

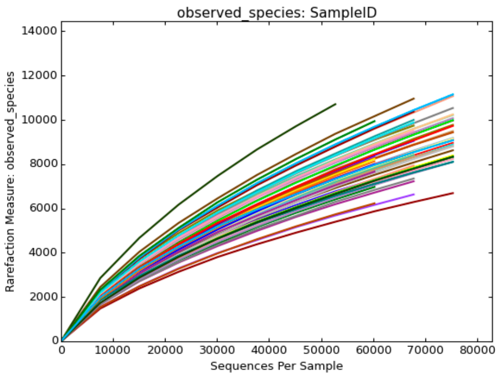

(c)

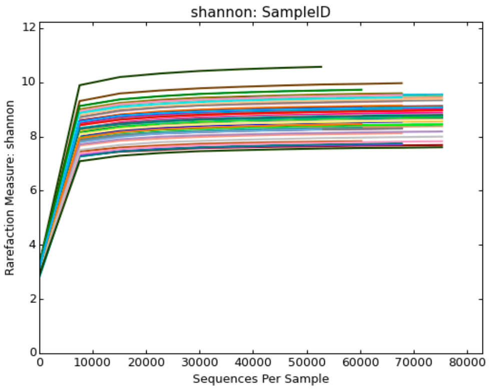

(d)

Figure S1. Alpha dilution curve of the sample; Note: a, b, c, d are PD, whole tree index, chao1 index, observed species index, shannon index dilution curve.

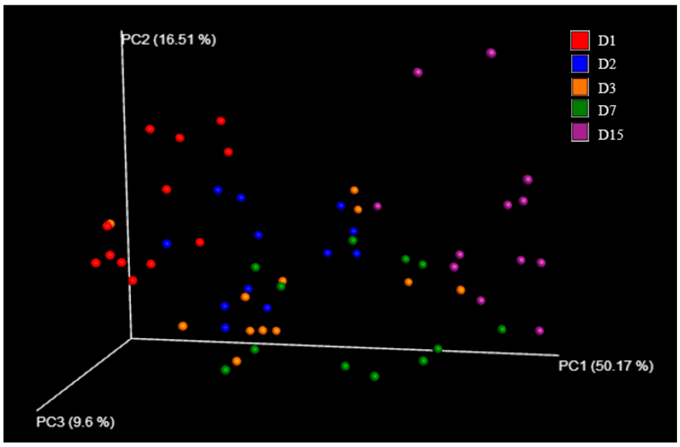

(a)

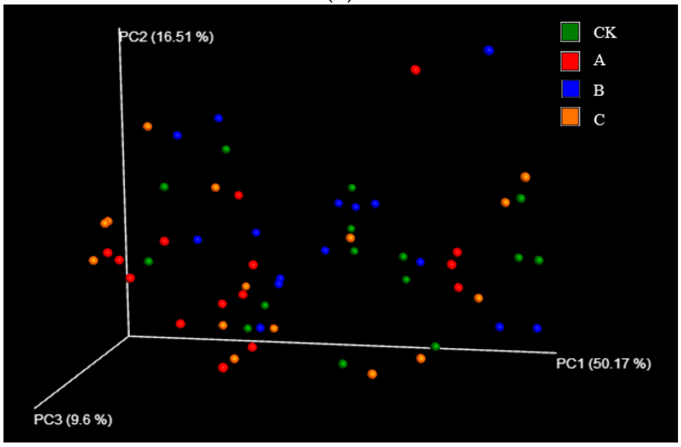

(b)

Figure S2. Sample cluster analysis; Note: (**a**), (**b**) are clusters analysis of samples, respectively, according to the days of composting and different treatment methods.

Table S1. Primary physicochemical characteristics of the raw materials (dry weight basis): laying hen manure, sawdust and biochar before composting.

	Moisture (%)	TOC (%)	TN (%)	C/N	pH	Surface area (m^2/g)	Pore volumes (cm^3/g)
**SB**	8.65	73.52	0.82	89.66			
**LM**	65.77	34.69	4.23	8.20	8.78	68.25	0.42
**SW**	8.54	52.85	0.31	170.48			

[1] SB: straw biochar; LM: laying hen manure; SW: sawdust; TN: total nitrogen (based on dry weight); TOC: total carbon (based on dry weight).

Table S2. Bacterial loading rate.

	Pre-loading bacteria content （CFU/mL）	Bacteria content after loading （CFU/mL）	Bacterial loading rate （%）
Bacillus stearothermophilus	2.14×10^8	7.89×10^6	96.31
Candida utilis	1.99×10^8	9.81×10^6	95.07
Bacillus subtilis	2.53×10^8	1.02×10^7	95.97
**Complex bacteria**	2.22×10^8	5.89×10^6	97.35

Table S3. The specific primers for bacteria, fungi, Bacillus stearothermophilus, Candida utilis, Bacillus subtilis.

Microorganism	Primer sequences（5′-3′）	Fragment size
**Bacteria**	F: ATTACCGCGGCTGCTGG	200bp
	R: CCTACGGGAGGCAGCAG	
**Fungi**	F: TCCGCAGGTTCACCTACGGA	340bp
	R: GAGGCAATAACAGGTCTG	
Bacillus stearothermophilus	F:ACGGGAGGCAGCAGTAGGGA	389bp
	R: GCCTTCGCCACTGGTGTTCC	
Candida utilis	F:TTACTACTTGGATAACCGTG	320bp
	R:GAGTCGCCTCCCGCCATAAG	
Bacillus subtilis	F: CCAACATCTCACGACACGAG	338bp
	R:AAGCAACGCGAAGAACCTTA	

Table S4. The ordinary PCR reaction program for bacteria, fungi, *Bacillus stearothermophilus*, *Candida utilis*, *Bacillus subtilis*.

Step	Bacteria	Fungi	*Bacillus stearothermophilus*	*Candida utilis*	*Bacillus subtilis*
**1.Initial denaturation**	94 °C, 4 min	94 °C, 5 min	95 °C, 3 min	95 °C, 2 min	95 °C, 3 min
**2. Denaturation**	94 °C, 30 s	94 °C, 60 s	95 °C, 30 s	95 °C, 2 min	95 °C, 30 s
**3. Primer annealing**	63 °C, 30 s	53 °C, 60 s	52 °C, 30 s	95 °C, 15 s	60 °C, 30 s
**4. Primer extension**	72 °C, 72 s	72 °C, 60 s	72 °C, 30 s	58 °C, 35 s	72 °C, 30 s
**5. Number of cycles**	35	35	34	40	35
**6. Final extension**	72 °C, 10 min	72 °C, 10 min	72 °C, 7 min	72 °C, 10 min	72 °C, 10 min
**7. Save**	4 °C	4 °C	4 °C	4 °C	4 °C

© 2020 by the authors. Licensee MDPI, Basel, Switzerland. This article is an open access article distributed under the terms and conditions of the Creative Commons Attribution (CC BY) license (http://creativecommons.org/licenses/by/4.0/).
